# Effect of different harvest times and processing methods on the vitamin content of Leymus

**DOI:** 10.3389/fnut.2024.1424334

**Published:** 2024-09-13

**Authors:** Yifan Wang, Xue Chen, Xingliang Zhuo, Lei Wang, Tingting Jia, Fangcai Ji, Hongrui Zhang, Zhu Yu

**Affiliations:** ^1^College of Grass Science and Technology, China Agricultural University, Beijing, China; ^2^School of Grassland Science, Beijing Forestry University, Beijing, China

**Keywords:** B vitamins, α-tocopherol, silage fermentation, natural drying, Leymus chinensis

## Abstract

**Objectives:**

The objective of this study was to investigate the effect of different harvest times and processing methods on the B vitamins and α-tocopherol contents of *Leymus chinensis* (Trin.).

**Methods:**

*L. chinensis* was harvested on 11 July (T_1_ group), 16 July (T_2_ group), 21 July (T_3_ group), 26 July (T_4_ group), and 31 July (T_5_ group) in 2022 and processed using natural drying and silage fermentation to evaluate fermentation quality, chemical composition, *in vitro* digestibility and vitamin content.

**Results:**

The fermentation quality of *L. chinensis* silage prepared at all five times of harvest was better. The silage fermentation group showed a significant increase (*p* < 0.05) in crude protein (CP), thiamin, riboflavin, pyridoxine and α-tocopherol content, a significant decrease (*p* < 0.05) in water-soluble carbohydrate (WSC) content, and small differences in neutral detergent fibre (NDF), acid detergent fibre (ADF), niacin and pantothenic acid content, when compared to the natural drying group. The content of thiamine, riboflavin, niacin, pantothenic acid and pyridoxine were higher in the pre-harvest period. In silage fermentation, the loss rate of thiamin, riboflavin and pyridoxine was positively correlated with pH and WSC, and the loss rate of thiamin and riboflavin was negatively correlated with lactic acid content. The loss rate of pantothenic acid was negatively correlated with pH and WSC, and positively correlated with lactic acid and ammonia nitrogen. The rate of α-tocopherol synthesis exceeded the rate of catabolism.

**Conclusion:**

The content of CP, thiamine, riboflavin, niacin, pantothenic acid and pyridoxine were higher during the early harvest period. Silage fermentation preserved the chemical composition and vitamin content of *L. chinensis* better than natural drying and had no effect on *in vitro* digestibility. During silage fermentation, the acidic environment promoted the preservation of thiamin, riboflavin and pyridoxine, but promoted the breakdown of pantothenic acid, α-tocopherol content increased through synthesis.

## 1 Introduction

*L. chinensis* is a perennial forage grass of the genus Lymus in the family Gramineae, *L. chinensis* represents over 50% of the forage production of natural grassland in the Inner Mongolian grasslands, and is a significant local forage resource. The hay of *L. chinensis* has excellent nutritional value, good palatability and high feeding rate, so at present, natural drying is the main way of utilising *L. chinensis* (1). The impact of varying harvest times on nutritional components, such as crude protein and neutral detergent fibre, in *L. chinensis* hay has been documented. Harvesting too early results in higher nutritional quality but lower yields, while harvesting too late increases the stem-to-leaf ratio of the forage, resulting in lower nutritional quality, higher fiber content and reduced forage intake (2). Nevertheless, the impact of varying harvest times on the vitamin content of *L. chinensis* hay has yet to be documented. B vitamins are a group of water-soluble vitamins that participate in the body’s metabolism as coenzymes or cofactors in ruminants. While, α-tocopherol is a general term for all tocopherols and tocotrienols with d-α-tocopherol activity, the main form of which is α-tocopherol, acting as a significant fat-soluble antioxidant in animals (3). Even ruminants are able to synthesise B vitamins through rumen microorganisms, while it is difficult to maintain the requirements of high productivity animals; they are able to synthesise α-tocopherol, neither. B vitamins and α-tocopherol still need to be supplemented through the intake of fresh or preserved forage (4). Dornbach et al. (5) concluded that supplementation of bulls with α-tocopherol increased their antioxidant capacity and reduced hair cortisol concentrations. Kanyar et al. (6) added α-tocopherol (400 mg/d) to lamb diets and found that it improved meat colour stability and lipid peroxidation in muscle tissue. Castagnino et al. (7) fed dairy cows with forages of different maturity levels and found that different types of forages at the same maturity level had different effects on the intake of B vitamins in cows. It is therefore of great importance to investigate the impact of varying harvest times on the vitamin content of *L. chinensis*.

Natural drying results in significant nutrient loss due to respiration during drying, if it rains during the drying process, it will exacerbate the loss of nutrients, resulting in a serious decline in feed quality. In comparison to hay, silage of *L. chinensis* has been demonstrated to reduce forage nutrient losses, improve digestibility, increase palatability and livestock intake, and also to improve some vitamin content (8). Harvest time is a crucial factor affecting forage quality. Czurgiel et al. (9) found that rams contained more α-tocopherol in their muscle tissue after feeding silage. Castagnino et al. (10) found that particle length of silage affected the efficiency of rumen synthesis of B vitamins in dairy cows. Van den Oever et al. (11) fed hay and silage separately to dairy cows and found that feeding silage increased B_12_ levels in milk relative to hay. Nevertheless, there is a paucity of research investigating the effects of B vitamins and vitamin E on *L. chinensis* silage harvested at different times, and the changing patterns and influencing factors in the silage process remain largely unexplored. Therefore, the aim of this study was to investigate the effects of different harvest times and processing methods on B vitamins and α-tocopherol, as well as to analyze the chemical composition and *in vitro* digestion.

## 2 Materials and methods

### 2.1 Test materials and experimental design

**Figure 1:**
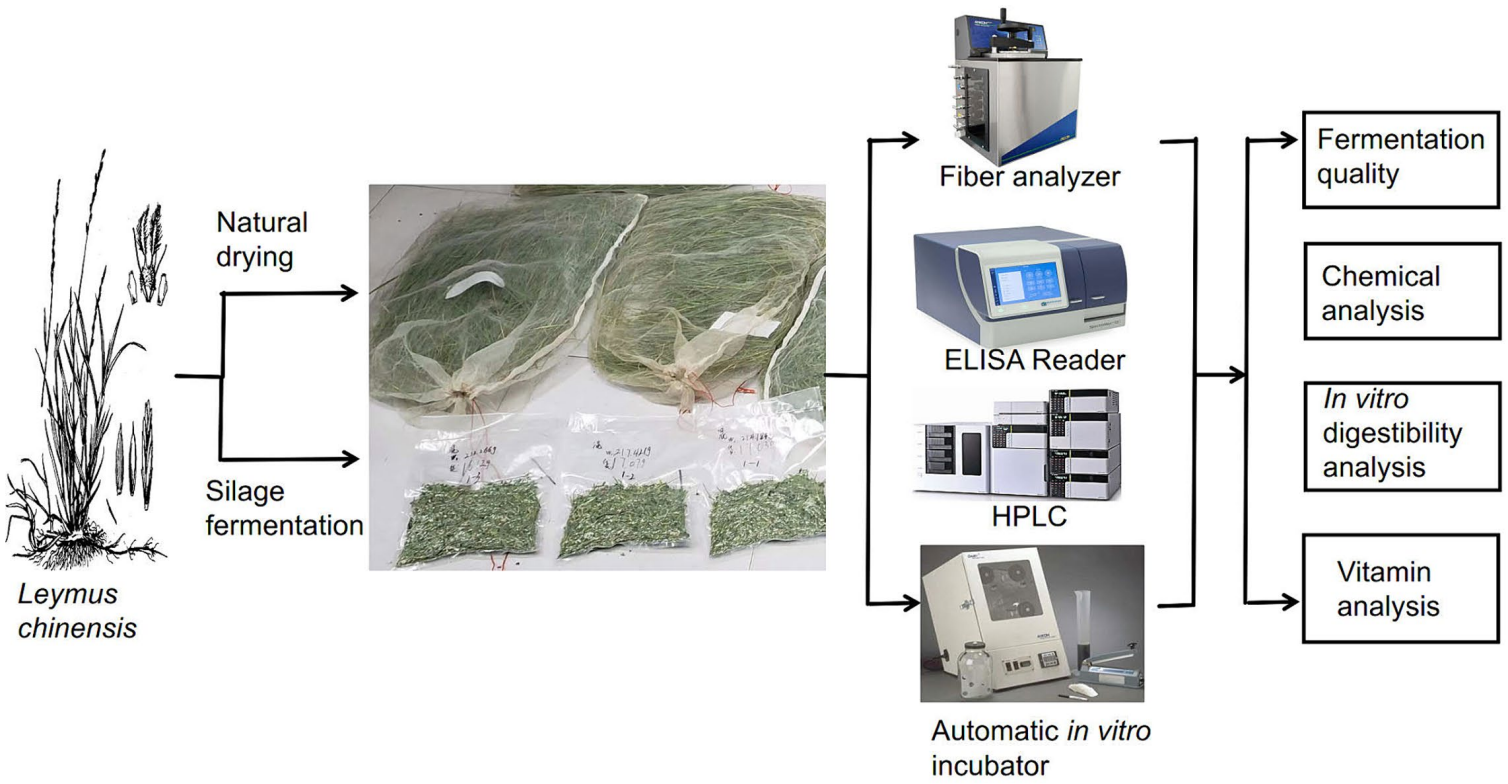
Flowchart of test operation. HPLC, high performance liquid chromatography.

The study area was located at the Grassland Agroecosystem Experimental Station in Chenbalhu Banner, Hulunbeier City, Inner Mongolia (N 49°20′–49°26′, E 119°55′–120°09′, altitude 628–649 m), P. R. China. and *Leymus chinensis* (Zhongke no. 2) (12) was harvested in the meadow grassland sample plots, using a five-point sampling method in 2022 on July 11 (T_1_ group), July 16 (T_2_ group), July 21 (T_3_ group), July 26 (T_4_ group), July 31 (T_5_ group). The processing methods of natural drying and silage fermentation were designed. Figure 1 provides the operational steps of the test. The specific methods of operation were as follows.

Natural drying group: *L. chinensis* was harvested with 4 to 5 cm stubble height. Approximately 500 g of *L. chinensis* was taken and placed in a nylon mesh bag to dry naturally in the sun until constant weight. Then chopped into about 2 cm pieces with a forage cutter, and then sampled. Each treatment had three repetitions.

Silage fermentation group: *L. chinensis* was harvested with 4 to 5 cm stubble height. The *L. chinensis* was chopped into about 2 cm piece with forage cutters. Then approximately 150 g of chopped *L. chinensis* forage were filled into a polyethylene bag silo (30 × 40 cm; 0.19 mm thickness) without any additives added. The silo was sealed with a vacuum sealer (FW3150; Fresh World Electir Co., Ltd., Guangzhou, China). Each treatment had three repetitions. All silos were stored at room temperature and opened and sampled after 70 days of ensiling.

### 2.2 Measurement content and methods

#### 2.2.1 Chemical analysis

Twenty-gram samples were mixed with 180 mL of distilled water and homogenized in a juicer for 2 min. The resulting mixture was filtered, and the filtrate was used to determine pH, ammonia nitrogen and organic acid content. The pH was measured using a pH meter (PHS-3C, INESA Scientific Instrument Co., Ltd., Shanghai, China). The levels of organic acids, including lactic, acetic, propionic, and butyric acids, were analysed using high-performance liquid chromatography (HPLC; column: Shodex RS Pak KC-811, Showa Denko KK, Kawasaki, Japan; detector: DAD, 210 nm, SPD-20A, Shimadzu Co., Ltd., Kyoto, Japan; eluent: 3 mmol/L HClO4, at a flow rate of 1.0 mL/min; column temperature: 50°C). The concentration of ammonia nitrogen was analysed using the phenol and sodium hypochlorite method (13). The dry matter (DM) content was measured after oven-drying at 65°C for 48 h. The oven-dried samples were milled and passed through a 0.425 mm screen to analyse conventional compositional of feeds, which included crude protein (CP), water-soluble carbohydrate (WSC), neutral detergent fiber (NDF), acid detergent fiber (ADF) and ether extract (EE). The CP content was analyzed according to the method provided by AOAC (14). The WSC content was analyzed by anthrone-sulphuric acid colourimetric method (15). The NDF and ADF contents were analyzed according to the procedures of Van Soest et al. (16).


$$\begin{array}{l} \mathrm{Dry}\;\mathrm{matter content of natural drying group}\left(\%\right)\\ =\mathrm{Weight of}\;\mathrm{hay}\;\mathrm{after drying}\;\mathrm{at}\;65^{{}^{\circ}}C/\\ \mathrm{Weight of}\;\mathrm{hay}\;\mathrm{before drying}\;\mathrm{at}\;65^{{}^{\circ}}C\times 100 \end{array}$$

$$\begin{array}{l} \mathrm{Dry}\;\mathrm{matter content of silage fermentation group}\left(\%\right)\\ =\mathrm{Weight of silage after drying}\;\mathrm{at}\;65^{{}^{\circ}}C/\\ \mathrm{Weight of silage before drying}\;\mathrm{at}\;65^{{}^{\circ}}C\times 100 \end{array}$$

$$\begin{array}{l} \mathrm{Dry}\;\mathrm{matter content of}\;\mathrm{raw}\;\mathrm{material}\left(\%\right)\\ =\mathrm{Weight of fresh grass after drying}\;\mathrm{at}\;65^{{}^{\circ}}C/\\ \mathrm{Weight of fresh grass before drying}\;\mathrm{at}\;65^{{}^{\circ}}C\times 100 \end{array}$$

#### 2.2.2 *In vitro* digestibility analysis

*In vitro* dry matter digestibility (IVDMD), *in vitro* neutral detergent fiber digestibility (IVNDFD) and *in vitro* crude protein digestibility (IVCPD) were determined by the following method: the oven-dried (65°C for 48 h) samples were milled and passed through a 0.425 mm screen, then 0.5 g was weighed accurately and placed in a fibre bag that had been soaked in acetone and naturally dried. The bag was put into the digestion tanks of the *in vitro* simulation incubator (Ankom Technologies, Macedon, NY, United States), with 24 samples and one blank control in each tank. 1,330 mL of A buffer and 266 mL of B buffer (Buffer A: 10 g KH_2_PO_4_, 0.5 g MgSO_4_-7H_2_O, 0.5 g NaCl, 0.1 g CaCl_2_-2H_2_O and 0.5 g urea dissolved in one litre of water. Buffer B: 15 g Na_2_CO_3_ and 1 g Na_2_S-9H_2_O dissolved in one litre of water) was added to the tanks, followed by 400 mL of filtered rumen fluid. Carbon dioxide (CO_2_) was then passed into the tanks until they were filled, and the lids were closed. Subsequently, the digestion jar was placed in an *in vitro* simulation incubator and digested at 39°C for 48 h. The samples were removed and rinsed with distilled water and dried in an oven at 65°C for 48 h (17).


$$\begin{array}{l} \mathrm{In}\;\mathrm{vitro}\;\mathrm{dry}\;\mathrm{matter digestibility}\;\left(\mathrm{IVDMD}\%\right)\\ =\left[\mathrm{Sample}\;DM-\left(\mathrm{Undigested}\;DM-\mathrm{Blank}\;DM\right)\right]/\\ \mathrm{Sample}\;DM\times 100 \end{array}$$

$$\begin{array}{l} \mathrm{In}\;\mathrm{vitro}\;\mathrm{crude protein digestibility}\;\left(\mathrm{IVCPD}\%\right)\\ =\left[\mathrm{Sample}\;CP-\left(\mathrm{UndigestedCP}-\mathrm{Blank}\;CP\right)\right]/\\ \mathrm{SampleCP}\times 100 \end{array}$$

$$\begin{array}{l} \mathrm{In}\;\mathrm{vitro}\;\mathrm{neutral detergent fiber digestibility}\;\left(\mathrm{IVNDFD}\%\right)\\ =\left(\mathrm{Sample}\;NDF-\mathrm{Undigested}\;NDF\right)/\\ \mathrm{SampleNDF}\times 100 \end{array}$$

#### 2.2.3 Vitamin analysis

The vitamin concentrations were determined by HPLC. The sample was freeze-dried using a vacuum freeze-drying machine for 2 days (FreeZone 4.5 L, LABCONCO Corp., Kansas City, MO, United States) before analysis. The freeze-dried samples were passed through a 0.425 mm screen.

Using the method of Jia et al. (18), thiamin, riboflavin, niacin, pantothenic acid, pyridoxine, and α-tocopherol were determined: thiamin concentrations were determined in duplicate based on the method of GB/T 14700-2018, riboflavin levels were analyzed in duplicate according to the method of GB/T 14701-2019, niacin levels were examined according to the method of GB/T 17813-2018, pantothenic acid levels were measured based on the method of GB/T 18397-2014, pyridoxine levels were determined based on the method of GB/T 14702-2018, the α-tocopherol levels were analyzed based on the method of GB/T 17812-2008.

### 2.3 Statistical analysis

The vitamin content, fermentation quality, *in vitro* digestibility, and chemical composition data were analyzed by analysis of variance (ANOVA), using the general linear model-univariate procedure of SPSS 19.0 software. ANOVAs were performed for harvest times and processing methods as the two main parameters and for the interaction between the two parameters. The mean values were compared using Duncan’ s multiplerange tests. Differences between means were considered significant when *p* < 0.05. Correlation analysis between the loss rate of vitamin B group or the growth rate of α-tocopherol in *L. chinensis* silage and other analyzed variables was performed using the Pearson correlation coefficient. Correlation analysis between the loss rate of vitamin B group or the growth rate of α-tocopherol in *L. chinensis* silage and other analyzed variables was performed using the Pearson correlation coefficient in SPSS (19).

## 3 Results and analysis

### 3.1 Chemical composition, vitamin content, and *in vitro digestibility* of *L. chinensis* before processing at different harvest times

Table 1 summarizes the chemical composition, vitamin content and *in vitro* digestibility of *L. chinensis* before processing at different harvest times. DM content was affected by harvest time (*p* < 0.001). The effect of harvest time on the chemical composition was significant (*p* < 0.05). The content of CP increased initially and then decreased, while the content of NDF and ADF decreased initially and then increased with increasing of harvest time. The T_3_ group had the lowest NDF, ADF, and EE content and the highest CP content. The effect of harvest time on the content of thiamine, riboflavin, niacin, pyridoxine and α-tocopherol was significant (*p* < 0.05) and insignificant (*p* > 0.05) on pantothenic acid content. The content of thiamine, riboflavin, niacin, pantothenic acid and pyridoxine increased initially and then decreased with the prolongation of the harvest time and then was maximum in the T_2_ group. The content of α-tocopherol increased initially and then decreased with increasing of the harvest time and then was maximum in the T_4_ group. There was no significant effect of harvest time on IVDMD and IVNDFD (*p* > 0.05), but there was a significant effect on IVCPD (*p* < 0.05). IVCPD increased initially and then decreased with increasing harvest time, with the highest value observed in the T_2_ group.

**Table 1:** Chemical composition, vitamin content, and *in vitro* digestibility of *L. chinensis* before processing at different harvest times.

Items	Harvest time					*p*-value
	T_1_	T_2_	T_3_	T_4_	T_5_	
Dry matter (%FW)	45.04 ± 0.40b	40.64 ± 0.23d	44.67 ± 0.18b	43.02 ± 0.30c	46.23 ± 0.19a	<0.001
Chemical composition (%DM)						
Crude protein	9.71 ± 0.39c	10.89 ± 0.50b	11.53 ± 0.37a	10.74 ± 0.14b	7.28 ± 0.21d	<0.001
Neutral detergent fiber	64.87 ± 0.38a	64.97 ± 0.21a	63.61 ± 0.80b	64.94 ± 0.17a	64.16 ± 0.39ab	0.014
Acid detergent giber	34.74 ± 0.26a	34.66 ± 0.27a	32.95 ± 0.24b	34.57 ± 0.15a	34.33 ± 0.26a	<0.001
Water-soluble carbohydrate	5.51 ± 0.28a	4.15 ± 0.32ab	4.22 ± 0.55ab	3.00 ± 0.44b	3.69 ± 1.50b	0.027
Ether extract	2.86 ± 0.12b	3.24 ± 0.60ab	2.67 ± 0.53b	4.05 ± 0.51a	3.95 ± 0.31a	0.011
Vitamins content (mg/kg DM)						
Thiamin	4.48 ± 0.48b	6.26 ± 0.62a	5.85 ± 0.32a	3.80 ± 0.62b	3.73 ± 0.41b	<0.001
Riboflavin	26.63 ± 2.06bc	39.91 ± 5.55a	33.16 ± 3.98b	26.52 ± 1.88bc	20.20 ± 3.17c	<0.001
Niacin	5.29 ± 0.89b	7.19 ± 0.37a	4.94 ± 0.35b	5.41 ± 0.51b	3.29 ± 0.68c	<0.001
Pantothenic acid	8.84 ± 0.75b	10.37 ± 1.03a	9.46 ± 0.34ab	8.78 ± 0.73b	9.15 ± 0.82ab	0.151
Pyridoxin	4.57 ± 0.07b	5.19 ± 0.50a	4.74 ± 0.24ab	3.57 ± 0.23c	3.24 ± 0.19c	<0.001
α-Tocopherol	108.08 ± 6.43c	103.27 ± 3.25c	106.39 ± 3.80c	165.13 ± 4.29a	133.23 ± 2.02b	<0.001
*In vitro* digestibility (%)						
*In vitro* dry matter digestibility	51.19 ± 4.88a	51.04 ± 3.05a	51.42 ± 6.21a	49.31 ± 5.44a	45.00 ± 3.94a	0.482
*In vitro* crude protein digestibility	58.33 ± 4.41ab	63.09 ± 4.48a	59.54 ± 3.19ab	55.99 ± 4.64ab	51.97 ± 2.70b	0.011
*In vitro* neutral detergent digestibility fiber	38.65 ± 5.94a	39.46 ± 4.34a	40.54 ± 7.14a	38.46 ± 3.04a	32.56 ± 3.34a	0.386

Different lowercase letters in the same line indicate significant differences between different harvesting times (*p* < 0.05), the same below.

### 3.2 Fermentation quality of *L. chinensis* silage at different harvest times

As shown in Table 2, harvest time significantly (*p* < 0.05) affected lactic acid, acetic acid, propionic acid, ammoniacal nitrogen content and pH for *L. chinensis silage*. The pH of the T_1_ group was significantly higher than that of the other groups (*p* < 0.05); the content of acetic acid in the T_1_ and T_3_ groups was significantly lower than that of the T_2_ and T_5_ groups (*p* < 0.05); the content of propionic acid in the T_2_ group was significantly higher than that of the other groups (*p* < 0.05); the content of butyric acid in the T_1_ group was significantly lower than that in the T_5_ group (*p* < 0.05), while the content of ammoniacal nitrogen in the T_5_ group was significantly higher than that in the other groups (*p* < 0.05).

**Table 2:** Fermentation quality of *L. chinensis* silage at different harvest times.

Items	T_1_	T_2_	T_3_	T_4_	T_5_	*p*-value
pH value	5.58 ± 0.01a	5.01 ± 0.03b	4.97 ± 0.49b	4.61 ± 0.03b	4.66 ± 0.14b	0.003
Lactic acid (%DM)	2.32 ± 0.18c	3.11 ± 0.06b	3.38 ± 0.23b	4.17 ± 0.26a	4.08 ± 0.12a	<0.001
Acetic acid (%DM)	0.48 ± 0.22b	1.50 ± 0.83a	0.40 ± 0.07b	1.01 ± 0.14ab	1.55 ± 0.15a	0.012
Propionic acid (%DM)	0.62 ± 0.42b	1.16 ± 0.27a	0.30 ± 0.07bc	0.24 ± 0.02bc	0.15 ± 0.01c	0.002
Butyric acid (%DM)	0.12 ± 0.06b	0.14 ± 0.06ab	0.18 ± 0.10ab	0.18 ± 0.06ab	0.26 ± 0.05a	0.201
Ammonia nitrogen (%TN)	3.43 ± 0.28d	5.15 ± 0.16c	3.72 ± 0.67d	6.82 ± 0.21b	7.81 ± 0.70a	<0.001

### 3.3 Chemical composition of *L. chinensis* forage with different harvest times and processing methods

Table 3 show that the interaction effect of harvest time and processing method on NDF, ADF, WSC and DM content was significant (*p* < 0.05). Harvest time and processing method had a significant (*p* < 0.05) effect on all indicators.

**Table 3:** Chemical composition of *L. chinensis* feed with different harvest times and processing methods.

Items	Processing method	Harvest period					*p*-value		
		T_1_	T_2_	T_3_	T_4_	T_5_	T	M	T*M
Dry matter (%FW)	Silage fermentation	48.88 ± 1.83aB	41.50 ± 0.09 dB	46.97 ± 0.43bB	44.48 ± 0.03cB	45.53 ± 0.23bcB	<0.001	<0.001	<0.001
	Natural drying	91.53 ± 1.12abA	91.11 ± 1.93abA	92.35 ± 0.02abA	93.92 ± 1.70aA	90.40 ± 1.73bA			
Crude protein (%DM)	Silage fermentation	7.68 ± 0.11bcA	9.00 ± 0.21aA	8.80 ± 0.23aA	7.77 ± 0.06bA	7.46 ± 0.10cA	<0.001	<0.001	0.586
	Natural drying	7.21 ± 0.22bB	8.19 ± 0.33aB	8.05 ± 0.12aB	7.12 ± 0.21bB	6.85 ± 0.20bB			
Neutral detergent fiber (%DM)	Silage fermentation	64.82 ± 0.96aA	64.39 ± 1.01aA	65.20 ± 0.08aA	65.41 ± 0.52aA	64.31 ± 1.09aA	0.006	<0.001	0.009
	Natural drying	64.74 ± 0.24aA	64.34 ± 0.27aA	62.17 ± 0.61bB	64.25 ± 0.67aA	62.26 ± 1.13bB			
Acid detergent giber (%DM)	Silage fermentation	35.11 ± 0.70abA	34.82 ± 0.41bA	34.72 ± 0.31bA	35.52 ± 0.42abA	36.01 ± 0.95aA	<0.001	<0.001	<0.001
	Natural drying	34.51 ± 0.39aA	34.43 ± 0.30aA	31.95 ± 0.41cB	34.52 ± 0.27aB	32.96 ± 0.89bB			
Water-soluble carbohydrate (%DM)	Silage fermentation	1.93 ± 0.27aB	1.09 ± 0.27cbB	1.14 ± 0.16bA	0.79 ± 0.18bcB	0.57 ± 0.39cB	0.003	<0.001	0.014
	Natural drying	3.23 ± 0.34aA	2.03 ± 0.52abA	1.65 ± 0.48bA	2.53 ± 1.10abA	3.11 ± 0.30aA			
Ether extract (%DM)	Silage fermentation	3.34 ± 0.58bA	3.39 ± 0.14bA	3.44 ± 0.59bA	4.49 ± 0.52aA	4.22 ± 0.42abA	0.002	<0.001	0.525
	Natural drying	2.96 ± 0.37abA	2.85 ± 0.12bB	2.52 ± 0.09bB	3.74 ± 0.29aB	2.99 ± 0.79abB			

Different uppercase letters in the same column indicate significant differences between different processing methods (*p* < 0.05). T, time effect; M, processing method effect; the interaction between T × M: the interaction between time and processing methods. The same below.

Within the silage fermentation group, the DM content was significantly higher (*p* < 0.05) in group T_1_ and significantly lower (*p* < 0.05) in group T_2_ than in the other harvest times; the CP content was highest in the T_2_ group, which did not differ significantly from the T_3_ group (*p* > 0.05), but was significantly higher than that of the T_1_, T_4_, and T_5_ groups (*p* < 0.05), while the T_5_ group had the lowest CP content; the NDF content of the groups did not differ significantly (*p* > 0.05); the content of ADF in the T_2_ and T_3_ groups did not differ significantly from that in the T_1_ and T_4_ groups (*p* > 0.05) and was significantly lower than that in the T_5_ group (*p* < 0.05); WSC content in T_1_ group was significantly higher than that in the other the harvest times (*p* < 0.05); and the EE content in T_4_ group was significantly higher than that of the T_1_, T_2_ and T_3_ groups (*p* < 0.05).

Within the natural drying group, the DM content of the T_4_ group was significantly higher than that of the T_5_ group (*p* < 0.05); the CP content of the T_2_ and T_3_ groups was significantly higher than that of the T_1_, T_4_ and T_5_ groups (*p* < 0.05); the NDF and ADF content of the T_3_ and T_5_ groups was significantly lower than that of the T_1_, T_2_ and T_4_ groups (*p* < 0.05), while the ADF content of the T_3_ group was significantly lower than that of the T_5_ group (*p* < 0.05); the WSC content of the T_3_ group was significantly lower than that of the T_1_ and T_5_ groups (*p* < 0.05), while the difference with T_2_ and T_4_ groups was not significant (*p* > 0.05); the EE content of the T_4_ group was significantly higher than that of the T_2_ and T_3_ groups (*p* < 0.05).

The CP content of silage fermentation group was significantly higher than that of natural drying group (*p* < 0.05); the NDF and ADF content of silage fermentation group was significantly higher than that of natural drying group in T_3_ and T_5_ groups (*p* < 0.05); the WSC content of silage fermentation group was significantly lower than that of natural drying group in T_1_, T_2_, T_3_ and T_4_ groups (*p* < 0.05); the EE content of silage fermentation group was significantly higher than that of natural drying group in T_2_, T_3_, T_4_ and T_5_ groups (*p* < 0.05).

### 3.4 *In vitro* digestibility of *L. chinensis* feed with different harvest times and processing methods

Table 4 shows that the interaction between harvest time and processing method did not have a significant effect (*p* > 0.05) on IVDMD, IVCPD, and IVNDFD. However, harvest time had a significant effect (*p* < 0.05) on IVNDFD, while processing method had a significant effect (*p* < 0.05) on IVDMD, IVCPD, and IVNDFD. In silage fermentation group, IVNDFD in T_4_ group was significantly higher than T_1_ and T_5_ groups (*p* < 0.05), and the difference of IVNDFD in T_2_, T_3_ and T_4_ groups was not significant (*p* > 0.05). In natural drying group, IVNDFD of group T_3_ was not significantly different from that of groups T_2_, T_3_ and T_4_ (*p* > 0.05) and was significantly higher than that of group T_1_ (*p* < 0.05). In T_5_ group, the silage fermentation group had significantly higher IVCPD (*p* < 0.05) and significantly lower IVDMD and IVNDFD (*p* < 0.05) compared to the natural drying group.

**Table 4:** *In vitro* digestibility of *L. chinensis* feed with different harvest times and processing methods (%).

Items	Processing method	Harvest period					*p*-value		
		T_1_	T_2_	T_3_	T_4_	T_5_	T	M	T × M
*In vitro* dry matter digestibility	Silage fermentation	43.48 ± 2.56aA	44.65 ± 1.15aA	46.81 ± 3.50aA	48.19 ± 2.80aA	45.57 ± 2.43aB	0.177	0.017	0.205
	Natural drying	45.52 ± 1.82aA	50.31 ± 5.82aA	51.98 ± 4.30aA	45.70 ± 4.63aA	51.89 ± 3.38aA			
*In vitro* crude protein digestibility	Silage fermentation	61.86 ± 7.93aA	66.90 ± 3.24aA	67.90 ± 5.69aA	67.31 ± 4.47aA	64.47 ± 3.61aA	0.158	0.001	0.912
	Natural drying	55.16 ± 5.30aA	63.31 ± 5.45aA	59.30 ± 3.75aA	60.80 ± 5.51aA	56.09 ± 3.20aB			
*In vitro* neutral detergent digestibility fiber	Silage fermentation	28.97 ± 1.94bA	30.39 ± 1.89abA	33.22 ± 3.67abB	35.61 ± 4.00aA	27.98 ± 1.98bB	0.027	0.010	0.167
	Natural drying	28.21 ± 6.21bA	36.89 ± 6.49abA	39.05 ± 3.04aA	35.55 ± 3.01abA	37.15 ± 4.09abA			

### 3.5 Vitamin content in *L. chinensis* feed with different harvesting times and processing methods

As shown in Table 5, the interaction effect of harvesting time and processing method on thiamin, riboflavin and α-tocopherol content was significant (*p* < 0.05); harvest time on all vitamin contents was significant (*p* < 0.05); processing method did not have significant effect on niacin content (*p* > 0.05), and the rest of the vitamins were significantly affected (*p* < 0.05).

**Table 5:** Vitamin content in *L. chinensis* feed with different harvesting times and processing methods.

Items	Processing method	Harvest period					*p*-value		
		T_1_	T_2_	T_3_	T_4_	T_5_	T	M	T*M
Thiamin (mg/kg DM)	Silage fermentation	1.44 ± 0.12bA	2.21 ± 0.19aA	2.08 ± 0.16aA	1.50 ± 0.15bA	1.59 ± 0.15bA	<0.001	<0.001	<0.001
	Natural drying	0.93 ± 0.05aB	1.06 ± 0.12aB	0.75 ± 0.08bB	0.41 ± 0.08cB	0.32 ± 0.04cB			
Riboflavin (mg/kg DM)	Silage fermentation	11.77 ± 0.64cA	19.91 ± 2.71aA	18.02 ± 1.99abA	15.04 ± 1.33bA	10.49 ± 1.35cA	<0.001	<0.001	<0.001
	Natural drying	3.82 ± 0.38aB	3.68 ± 0.25aB	3.45 ± 0.14aB	3.65 ± 0.86aB	3.05 ± 0.88aB			
Niacin (mg/kg DM)	Silage fermentation	3.50 ± 1.02abA	4.52 ± 0.75aA	2.95 ± 0.16bA	3.37 ± 0.82abA	2.25 ± 0.67bA	0.001	0.940	0.924
	Natural drying	3.49 ± 0.89abA	4.34 ± 0.55aA	3.09 ± 0.56bA	3.01 ± 0.38bA	2.56 ± 0.67bA			
Pantothenic acid (mg/kg DM)	Silage fermentation	4.78 ± 0.42abA	5.43 ± 0.87aA	4.48 ± 0.63abA	3.81 ± 0.82bA	3.89 ± 0.80bA	0.019	0.002	0.469
	Natural drying	3.55 ± 0.44aB	4.07 ± 0.78aA	4.06 ± 0.44aA	3.61 ± 0.37aA	3.00 ± 0.57aA			
Pyridoxing (mg/kg DM)	Silage fermentation	1.78 ± 0.29bcA	2.16 ± 0.18aA	1.99 ± 0.10abA	1.62 ± 0.17cA	1.61 ± 0.18cA	0.001	<0.001	0.151
	Natural drying	0.90 ± 0.03bB	1.04 ± 0.12aB	0.92 ± 0.09abB	0.87 ± 0.06bB	0.83 ± 0.04bB			
α-Tocopherol (mg/kg DM)	Silage fermentation	166.05 ± 3.19cA	156.99 ± 6.56cA	166.39 ± 3.97cA	244.72 ± 10.55aA	204.52 ± 10.07bA	<0.001	<0.001	<0.001
	Natural drying	40.67 ± 4.07bB	39.96 ± 2.11bB	42.41 ± 3.41bB	57.33 ± 3.71aB	53.36 ± 3.47aB			

In silage fermentation group, the contents of thiamine, riboflavin, and pyridoxine initially increased and then decreased as the harvest time was prolonged, while the T_2_ group had significantly higher levels of these vitamins compared to the T_1_, T_4_, and T_5_ groups (*p* < 0.05), but there was no significant difference between the T_2_ and T_3_ groups (*p* > 0.05). The T_2_ group had the highest niacin content, which was significantly higher than the T_3_ and T_5_ groups (*p* < 0.05). The pantothenic acid content of the T_2_ group was significantly higher than T_4_ and T_5_ groups (*p* < 0.05); The α-tocopherol content initially increased and then decreased as the harvest time was prolonged, while the highest content was observed in the T_4_ group, which was significantly higher than the other groups (*p* < 0.05). Additionally, the T_5_ group also had significantly higher α-tocopherol content than the T_1_, T_2_, and T_3_ groups (*p* < 0.05).

In natural drying group, the T_2_ group had significantly higher levels of thiamine and niacin compared to the T_3_, T_4_, and T_5_ groups (*p* < 0.05), and the difference with the T_1_ group was not significant (*p* > 0.05). pyridoxine content was significantly higher in the T_2_ group than in the T_1_, T_4_ and T_5_ groups (*p* < 0.05); and α-tocopherol content in the T_4_ and T_5_ groups was significantly higher than in the T_1_, T_2_ and T_3_ groups (*p* < 0.05).

Thiamin, riboflavin, pyridoxine and α-tocopherol content was significantly higher (*p* < 0.05) in silage fermentation group than in natural drying group.

### 3.6 Pearson correlation analysis between the loss rate of vitamin B group or the growth rate of α-tocopherol in *L. chinensis* silage and other analyzed variables

As shown in Table 6. In *L. chinensis* silage, the rate of thiamin loss was positively correlated with pH and WSC (*p* < 0.05), and negatively correlated with lactic acid, propionic acid, ammoniacal nitrogen, and ADF (*p* < 0.05); the rate of riboflavin loss was positively correlated with pH and WSC (*p* < 0.05), and negatively correlated with lactic acid, EE and IVCPD (*p* < 0.05); the rate of pyridoxine loss was positively correlated with pH and WSC (*p* < 0.05), and negatively correlated with propionic acid and ammoniacal nitrogen (*p* < 0.05); the rate of pantothenic acid loss was positively correlated with lactic acid and ammoniacal nitrogen (*p* < 0.05), and negatively correlated with pH and WSC (*p* < 0.05); There was no correlation found between the rate of niacin loss and the other variables (*p* > 0.05); The growth rate of α-tocopherol was negatively correlated with IVCPD (*p* < 0.05).

**Table 6:** Pearson correlation analysis between the loss rate of vitamin B group or the growth rate of α-tocopherol in *L. chinensis* silage and other analyzed variables.

Variable	Thiamin (mg/kg DM)	Riboflavin (mg/kg DM)	Niacin (mg/kg DM)	Pantothenic acid (mg/kg DM)	Pyridoxing (mg/kg DM)	α-Tocopherol (mg/kg DM)
Dry matter	0.250	0.332	0.003	−0.076	0.070	0.267
pH	0.693**	0.600*	−0.139	−0.542*	0.633*	0.408
Chemical composition (%DM)						
Lactic acid	−0.768**	−0.763**	−0.003	0.754**	−0.501	−0.140
Acetic acid	−0.451	−0.088	−0.214	0.243	−0.319	0.037
Propionic acid	0.465	0.439	0.193	−0.488	0.251	0.077
Butyric acid	−0.629*	−0.326	−0.239	0.252	−0.688**	0.100
Ammonia nitrogen	−0748**	−0.441	−0.134	0.615*	−0.561*	−0.297
Crude protein	0.357	−0.094	0.251	−0.336	0.299	0.212
Neutral detergent fiber	0.192	−0.239	0.279	0.375	−0.016	−0.031
Acid detergent fiber	−0.558*	0.066	0.327	0.364	−0.500	0.080
Water-soluble carbohydrate	0.741**	0.557*	−0.116	−0.535*	0.519*	0.107
Ether extract	−0.354	−0.546*	−0.318	0.463	−0.279	−0.151
*In vitro* digestibility						
*In vitro* dry matter digestibility	−0.390	−0.337	0.300	0.141	−0.041	−0.055
*In vitro* crude protein digestibility	0.022	−0.522*	−0.052	0.168	−0.332	−0.558*
*In vitro* neutral detergent fiber digestibility	−0.019	−0.384	0.269	0.108	0.084	−0.299

*95% confidence level and **99% confidence level.

## 4 Discussion

### 4.1 Effect of different harvest times on the fermentation quality of *L. chinensis* silage

It is erroneous to assume that pH is the sole criterion for evaluating the quality of fermentation. A multitude of factors, including dry matter content and buffering energy, exert a profound influence on the pH of silage (17). The pH values of *L. chinensis* silage at different harvest times were higher than 4.2, but the NH3-N/TN values were lower than 8%, while the fermentation quality was still good. The low moisture content of *L. chinensis* silage inhibits the growth of moulds and spoilage bacteria such as clostridia, which hinders the production of butyric acid and the decomposition of proteins (17). Additionally, the anaerobic state inhibits the activity of fungi. The activity of lactic acid bacteria is also weakened by low moisture content, resulting in fewer organic acids being produced and a higher pH value (20).

### 4.2 Effects of different harvest time and processing methods on the chemical composition of *L. chinensis*

The cell wall will be degraded into soluble monosaccharides during silage (65%–75% moisture content), and the content of NDF and ADF will be reduced to provide carbon source for lactic acid bacteria to improve silage quality (21). The NDF and ADF contents of silage fermentation group in this experiment are not different or even higher than those of natural drying group, this is because the moisture content of *L. chinensis* silage is low, and the osmotic pressure of plant cells reaches 55 × 10^5^–60 × 10^5^ Pa (22) at low moisture, in this case, the life activities of spoilage bacteria and lactic acid bacteria were close to physiological drying, which restricted their growth and reproduction. This also led to a reduction in the enzyme responsible for fiber degradation, resulting in a decrease in fiber decomposition. As a result, the content of NDF and ADF did not decrease after silage. In the silage fermentation group, the NDF and ADF content increased relatively due to the decrease in WSC content. The WSC content of the silage fermentation group was lower than that of the natural drying group. This is because lactic acid bacteria consume soluble carbohydrates during the silage process, leading to a decrease in WSC content after silage. As the plant growth time increases, the ratio of stem to leaves gradually increases, while the content of CP decreases and the content of NDF and ADF increases (23). But in this experiment, the CP content initially increased and then decreased with the prolongation of harvest time. Similarly, the NDF and ADF content fluctuated with the prolongation of harvest time. These changes may be attributed to the significant correlation between the growth state of forage grasses and environmental factors such as temperature, precipitation, humidity, and length of light exposure (24).

### 4.3 Effect of different harvest times and processing methods on *in vitro* digestibility

IVNDF in groups T_3_ and T_5_ and IVDMD in group T_5_ were lower in silage fermentation than in natural drying, which is consistent with the study by Riberio et al. (25). This is due to the fact that a greater loss of WSC occurs during the ensiling process, whereas a lesser loss of WSC takes place during the natural drying process. Following the loss of WSC, the NDF content expressed as a percentage of DM increased, resulting in an elevated NDF content after ensilage. WSC is readily digestible during *in vitro* digestion, resulting in a notable increase in IVDMD in natural drying group. Similarly, the ensiling process generates enzymes and acidic conditions that facilitate the degradation of hemicellulose, whereas the natural drying process does not degrade hemicellulose. Given that hemicellulose is an easily digestible component of the fiber composition, the readily digestible fiber components in silage decline, resulting in an increase in IVNDF in the silage fermentation group. Enzymatic in silage can alter its surface structure, increasing porosity, surface area, and accessibility between fibers and crude proteins, enhancing the contact area between rumen microbial enzymes and silage in ruminants (26). Simultaneously, the enzyme cleaves the bond connecting cellulose and hemicellulose, reducing the crystallinity of cellulose and increasing its specific surface area. This facilitates the attachment and degradation of rumen microorganisms (27). Thus, there were no significant differences in IVDMD and IVNDF between the silage fermentation group and the natural drying group during other times.

### 4.4 Effect of different harvest time and processing methods on vitamin content

The pre-harvest period showed higher levels of thiamine, riboflavin, niacin, pantothenic acid, and pyridoxine, which is consistent with the findings of Castagnino and Tadevosyan (7, 28). It is currently believed that B vitamins in plants are synthesized in chloroplasts, and the number of chloroplasts is directly related to the content of B vitamins. Leaves are richer in chloroplasts compared to stems. Early in the life of the plant, the stem-leaf ratio is low, with more chloroplasts per unit of mass. As the harvest time was prolonged, the stem-leaf ratio of the plant was elevated. Consequently, in the later stages of the plant, B vitamin content declined (23).

Silage fermentation effectively reduced the loss of thiamin, riboflavin, and pyridoxine during storage compared to natural drying. This is because these vitamins are highly sensitive to light and easily oxidised by oxygen, however, they are relatively stable to heat and oxygen under acidic conditions (29). Thiamine, riboflavin, and pyridoxine undergo oxidation and photodegradation during prolonged natural drying, but the acidic, anaerobic, light-avoiding environment of silage fermentation reduced the oxidation and photodegradation of these vitamins (30). However, a study by van den Oever et al. (11) found no significant difference in thiamin levels between silage and sun-drying treatments. This may be due to the instability of thiamin, which could degrade during the silage process if exposed to direct sunlight or excess oxygen in the pre-silage stage. In silage, the rates of thiamin, riboflavin and pyridoxine loss were positively correlated with pH and WSC, and thiamin and riboflavin loss rates were negatively correlated with lactic acid content. Due to the similar characteristics of thiamin, riboflavin, and pyridoxine (29), as well as the fact that the rate of loss of pyridoxine is independent of the lactate content, we cannot infer that lactate affects the rate of loss of thiamin and riboflavin, despite the negative correlation between the rate of loss of thiamin and riboflavin and the lactate content. As lactic acid production from WSC consumption by *Lactobacillus* in silage fermentation leaded to a decrease in pH, it was initially deduced that pH is the main factor affecting the rates of loss of thiamin, riboflavin and pyridoxine. The pH was reduced by lactic acid production by *Lactobacillus* to slow down the rates of loss of thiamin, riboflavin and pyridoxine in silage. The lacked of correlation between the rate of pyridoxine loss and lactic acid content may be attributed to the superior stability of pyridoxine in comparison to thiamine and riboflavin (31).

Niacin is a water-soluble vitamin that is highly stable and tolerant to oxygen, acid, heat, and light in both aqueous and solid systems (32). One study indicated that niacin-rich bakery products experienced only a slight decrease in niacin content after 8 months of storage, regardless of storage conditions (with or without light and with or without refrigeration) (33), however, niacin was still lost to varying degrees (1–12%) at different high temperatures (34). Jia et al. (18) discovered that the niacin content of *L. chinensis* decreased by more than 50% after silage, and that there was further loss of niacin content after the addition of lactobacilli. The results of the present experiment were consistent with those of the above experiments. There were varying degrees of loss in niacin content in *L. chinensis* after undergoing silage fermentation and natural drying, but there was no significant difference in the niacin content between the two processes. This could be attributed to the fact that niacin has a high tolerance to oxygen, acid, heat and light, while the effect of oxygen and light on niacin was minimal in the case of sun-drying, which led to the absence of differences.

In silage, the rate of pantothenic acid loss was negatively correlated with pH and WSC, and positively correlated with lactic acid and ammoniacal nitrogen. Consumption of WSC by lactobacilli, which produced lactic acid, affected pH and the rate of pantothenic acid loss, suggesting a pathway for pantothenic acid loss. The rate of loss of pantothenic acid decreased with an increase in pH. Pantothenic acid is more stable in weakly alkaline environments than in acidic ones. It is hydrolysed in acidic environments. The highest stability of pantothenic acid is between pH 5–7. As the pH content of T_1_ and T_2_ groups was greater than 5, the loss of pantothenic acid was lower in T_1_ and T_2_ groups than in other groups (35).

The primary active constituent of α-tocopherol is α-tocopherol. It is present in the chloroplasts of plants and is produced in two ways within the plant body. One method involves synthesising it from scratch through chloroplasts, while the other involves phytol, a precursor of α-tocopherol synthesis, which is produced directly through the degradation of chloroplasts (36). The increase in α-tocopherol content during silage could be attributed to the degradation of chloroplasts, which produced large amounts of phytol. Phytol synthesised α-tocopherol, and the rate of synthesis was much greater than the rate of α-tocopherol degradation (37). The increase in α-tocopherol content in the T_4_ group compared to the previous three periods may be attributed to the large number of spikes in the *L. chinensis* and the α-tocopherol content in the spikes was higher than that in the stems and leaves, resulting in an overall increase in α-tocopherol content (38). The content of α-tocopherol was significantly reduced after natural drying due to its sensitivity to light and oxygen (39). Hidiroglou et al. (40) found that the α-tocopherol content of hay was 30–40% of that of silage. In this study, *L. chinensis* hay had a slightly higher α-tocopherol content, which may be related to the climatic conditions during sun-drying. In addition, it was found that the acidic environment is very favorable for the preservation of α-tocopherol and promotes the release of Mg^+^ to synthesize α-tocopherol. The lowest loss of α-tocopherol occurred at pH 4, with an increase in pH resulting in a higher loss of α-tocopherol (41). However, the growth rate of α-tocopherol in silage in this experiment was not correlated with pH and other variables. This may be because the rate of α-tocopherol synthesis in silage exceeded the rate of degradation, which is consistent with the findings of Tian et al. (42).

## 5 Conclusion

The silage made from *L. chinensis* exhibited good fermentation quality at all harvest times. Furthermore, the silage fermentation preserved the chemical component and vitamin content of the *L. chinensis* better than natural drying. Levels of crude protein (CP), thiamin, riboflavin, niacin, pantothenic acid and pyridoxine were higher during the early harvest period. During silage fermentation, the acidic environment promoted the preservation of thiamin, riboflavin, and pyridoxine, but it also accelerated the decomposition of pantothenic acid. In contrast, niacin was relatively stable during processing. Meanwhile α-tocopherol content was increased.

## Data Availability

The original contributions presented in the study are included in the article/supplementary material, further inquiries can be directed to the corresponding author.
